# Reply to Same

**Published:** 1883-10

**Authors:** C. S. Carr


REPLY BY DR. CARR.

My reviewer of the present number of THE BISTOURY very facetiously comes forward with an article composed mainly, if not entirely of misapprehensions of the article reviewed, as well as the *subject* to which the article relates. Without further preface, we will refer to them in the order in which they occur:

1st. That an attack on infinitesimalism is to be construed to be an attack on homœopathy. Infinitesimalism is no more a part of homœopathy than the Nebular hypothesis is a part of Calvinism.

2d. That the second law quoted in the reviewed article is not to be found in the "New Chemistry." It is not only to be found in the "New Chemistey" referred to, but in every chemistry of recent date.

3d. That any series of numbers increasing by multiples is arithmetical progression, instead of geometrical. Of course, the above should be reversed.

4th. That there is any such thing as a "permanent gas." The term "permanent gas" is obsolete, the last known gas having been conquered.

5th. That steam is not a proper gas. Upon this error we can offer no comment, save to assert the contrary, and express our astonishment that such a proposition should have found its way into print.

*Any gas* reduced to a maximum of density is a *vapor; any vapor* above the maximum of density is a *gas*. So much for the misapprehensions of the *subject* under discussion. And now we will briefly refer to two serious misapprehensions of the article itself:

1st. That it was intimated, directly or indirectly, that the *gas steam* could be reduced to a temperature of 32° F. without changing its gaseous form, or that it would in any particular subserve the purposes of the article if it could be so reduced.

2d. That the whole argument contained in the article is based on the assumption that it had been proved that a cubic inch of steam contained one septillion molecules.

The assumption was that it had been shown that a cubic inch of steam could not, according to the laws quoted, contain *more* than one septillion molecules, but the unavoidable inference was that it contained *far less*; but undoubtedly the article lacked the amount of clearness necessary to convey the intention of the author. We will briefly go over the same ground again in a slightly different manner:

Suppose I have two vials, each containing a cubic inch of gas—one hydrogen, the other steam; the hydrogen at a temperature of 32° F. and at a pressure of one atmosphere, or 30 in. barometer, but in the case of the steam the temperature will have to be 212° F. with the same pressure. In the case of the cubic inch of hydrogen, it is shown that it contains one septillion molecules, by the second law, which was quoted *verbatim* from a reliable chemistry, viz: "The number of molecules in a cubic inch," etc. I must now produce like conditions in the gases before I can use the first law, which I do by raising the temperature of the hydrogen and *not* by the silly attempt of reducing the temperature of the steam. But I cannot raise the temperature of the hydrogen without diffusing it through more space, unless I increase the pressure; and, as we must not change the pressure if we are to produce like conditions, we will be obliged to diffuse it through greater space; or in other words, by the time we have raised the temperature from 32° F. to 212° F. (the temperature of the steam) we will have in each cubic inch of the hydrogen gas less than one septillion molecules—just how many less is easily computed from the amount of expansion the hydrogen undergoes. Then, with one cubic inch of our rarified hydrogen and a cubic inch of steam reduced to like conditions, we quote the first law, or law of Avogadro, viz: Equal volumes of *all substances when raised to a state of gas* under like condition, contain an *equal number of molecules.*

If our steam contains an equal number of molecules, and our hydrogen contains less than one septillion, then, of course, it follows that the cubic inch of steam contains less also. If experience is to be of any value as a teacher, every vestige of error must be eliminated from our interpretation of the facts which go to make up our experiences.

THE STIMULATING PROPERTIES OF OATS.—In view of the extraordinary claims which have during the past year or so been made for a preparation manufactured from oats, the results of certain experiments made by M. A. Sanson, and presented to the *Academie des Sciences*, will be read with interest. The experimenter tested by means of Dubois Reymond's apparatus the neuro-muscular excitability of horses before and after the exhibition of oats, and as a result of his experiments we have the following conclusions:

1. The pericarp of the oat contains a substance soluble in alcohol, which has the property of stimulating the motor as well as the nervous system.

2. This substance is nitrogenous, and probably belongs to the group of the alkaloids. It is uncrystallizable, brownish, finely granular. Its formula (subject to verification) is $C_{50}$, $H_{21}$, $NO_{18}$. It is called avenine.

3. All varieties of oats contain some of this alkaloid, but to an unequal extent, the difference depending upon the soil as well as variety. The black variety of the grain contains generally the most.

4. If the amount of avenine is below 9-10 per cent. of dried oats, it is insufficient to cause excitation. No facts are given regarding the effect of avenine upon the human organism.

BLEACHING SPONGES.—The following process was, says *New Remedies*, devised by Mr. John Borham, and has been in use in Bellevue Hospital for a considerable time:

Soak the sponges, previously deprived of sand and dirt by beating and washing, in a one per cent. solution of permanganate of potassium. Then remove them, wash them thoroughly with water, and press out the water. Next, put them into a solution of one-half pound of hyposulphite of sodium in one gallon of water, to which one ounce of oxalic acid has been added, and leave them in the solution for fifteen minutes. Finally, take them out, and wash them thoroughly.

By this treatment the sponges are rendered perfectly white. Many sponges contain a more or less dark-colored, brownish core. If treated only with permanganate and acid, the core is either not bleached at all, or if it has been somewhat bleached, the tint is apt to grow again darker. By the above modification, every portion of the sponge is rendered white, and remains so.

Send in your subscriptions for the present year.

